# White matter tract alterations in schizophrenia identified by DTI-based probabilistic tractography: a multisite harmonisation study

**DOI:** 10.1017/neu.2024.2

**Published:** 2024-02-13

**Authors:** Young Tak Jo, Sung Woo Joo, Woohyeok Choi, Soohyun Joe, Jungsun Lee

**Affiliations:** 1 Department of Psychiatry, Kangdong Sacred Heart Hospital, Hallym University College of Medicine, Seoul, Korea; 2 Department of Psychiatry, Asan Medical Center, University of Ulsan College of Medicine, Seoul, Korea; 3 Brain Laboratory in the Department of Psychiatry, School of Medicine, University of California, San Diego, CA, USA

**Keywords:** schizophrenia, DTI, harmonisation, probabilistic tractography, multisite study

## Abstract

**Introduction:**

It has been suggested that schizophrenia involves dysconnectivity between functional brain regions and also the white matter structural disorganisation. Thus, diffusion tensor imaging (DTI) has widely been used for studying schizophrenia. However, most previous studies have used the region of interest (ROI) based approach. We, therefore, performed the probabilistic tractography method in this study to reveal the alterations of white matter tracts in the schizophrenia brain.

**Methods:**

A total of four different datasets consisted of 189 patients with schizophrenia and 213 healthy controls were investigated. We performed retrospective harmonisation of raw diffusion MRI data by dMRIharmonisation and used the FMRIB Software Library (FSL) for probabilistic tractography. The connectivities between different ROIs were then compared between patients and controls. Furthermore, we evaluated the relationship between the connection probabilities and the symptoms and cognitive measures in patients with schizophrenia.

**Results:**

After applying Bonferroni correction for multiple comparisons, 11 different tracts showed significant differences between patients with schizophrenia and healthy controls. Many of these tracts were associated with the basal ganglia or cortico-striatal structures, which aligns with the current literature highlighting striatal dysfunction. Moreover, we found that these tracts demonstrated statistically significant relationships with few cognitive measures related to language, executive function, or processing speed.

**Conclusion:**

We performed probabilistic tractography using a large, harmonised dataset of diffusion MRI data, which enhanced the statistical power of our study. It is important to note that most of the tracts identified in this study, particularly callosal and cortico-striatal streamlines, have been previously implicated in schizophrenia within the current literature. Further research with harmonised data focusing specifically on these brain regions could be recommended.

Significant Outcomes:

- We utilised probabilistic tractography with diffusion MRI data, offering an alternative methodological approach that complements the deterministic tractography’s limitations.
- By merging datasets from multiple study sites and applying harmonisation methods, we significantly enhanced our statistical power and adjusted for inter-site image variabilities, ensuring more reliable and generalisable results.
- We revealed significant alterations in various brain white matter tracts in schizophrenia patients and further elucidated their relationship with cognitive measures.

Limitations:

- Our study did not encompass some demographic variables, such as ethnicity, education attainment, and socioeconomic status, constraining a more comprehensive understanding of factors influencing white matter integrity in schizophrenia.
- We did not account for certain clinical variables, such as current clinical status, illness duration, or the potential influence of antipsychotic medications, which could affect the observed relationships and results.

## Introduction

Schizophrenia is a chronic, debilitating psychiatric disorder characterised by hallucination, delusion, and disorganised behavior. It has been recognised as a severe mental illness that results in a significant global disease burden (Charlson *et al*., 2018). Therefore, there has been much research focusing on the pathophysiology of schizophrenia, including various imaging studies (Karlsgodt *et al*., 2010; Jo *et al*., 2020; Joo *et al*., 2021a; 2021b). As the name schizophrenia refers to, it has been suggested that schizophrenia involves the disintegration of connectivity between functional brain regions (Friston *et al*., 2016, Schmitt *et al*., 2011).

The diffusion tensor imaging (DTI), which allows for in vivo examination of white matter microstructures, has widely been used for studying the brain with schizophrenia (Kubicki *et al*., 2007; Lee *et al*., 2013). Specifically, tractography, which builds a three-dimensional model from DTI, has been a promising investigation method to visualise nerve tracts within the brain (Yamada *et al*., 2009) and explore the connectivity between functional regions. Nevertheless, most studies have used the region of interest (ROI) based approach, which relies on only one of the tractography methods, the deterministic tractography. Deterministic tractography has been widely used in the study of the brain, particularly in patients with schizophrenia, due to its relative simplicity and time-efficient image acquisition procedures.

However, there are limitations to deterministic tractography that may hinder its accuracy and ability to fully characterise the brain’s white matter microstructures. One of the primary limitations of deterministic tractography is its reliance on a single diffusion orientation per voxel. Considering that the voxel size of around 2–3 mm per dimension in diffusion-weighted imaging acquisitions is usually larger than the 1 mm diameter of fibre bundles, using only one diffusion orientation may not adequately capture the complexity of the brain’s white matter tracts. This limitation can lead to false tracts or the tracking process stopping in regions with isotropic tensors (Descoteaux *et al*., 2009). Specifically, some voxels containing crossing, branching, or fanning fibres or bottleneck formations are not adequately represented by deterministic tractography (Jones, 2010). Furthermore, deterministic tractrography can follow false tracts or may stop in regions with isotropic tensors.

Probabilistic tractography, on the other hand, differs from deterministic tractography in that it estimates the diffusion parameters’ distribution at each voxel using statistical techniques (Parker & Alexander, 2003; 2005). By repeating thousands of statistical samplings, researchers can evaluate the existing probabilities of streamlined brain tracts with diffusion measures (Price *et al*., 2008; Shon *et al*., 2018). As a result, probabilistic tractography is generally more sensitive in detecting fibres than deterministic tractography, particularly in regions with low anisotropy (Fillard *et al*., 2011). It can therefore offer a more comprehensive assessment of white matter connectivity by considering uncertainties in the diffusion data and generating a distribution of possible fibre pathways, thus reducing potential for subjective bias and providing a more robust evaluation of white matter alterations in schizophrenia (Behrens *et al*., 2007; Descoteaux *et al*., 2009).

Therefore, we tried to use probabilistic tractography to overcome the limitation of deterministic tractography. In fact, few previous studies have explored the potential value of probabilistic fibre tracking for studying white matter microstructures in schizophrenia (Khalsa *et al*., 2014; Cho *et al*., 2016; Schlaier *et al*., 2017), with most focusing on disrupted thalamo-cortical connectivity. Furthermore, we utilised data from different study sites, including public data, to overcome sample size limitations. In fact, it has been suggested that a multicentre study including a large number of brain image samples is required (Pearlson, 2009; Van Horn & Toga, 2009). The recent ENIGMA study indeed involved many study participants recruited from different centres (Kelly *et al*., 2018). Our hypothesis is that using probabilistic tractography in combination with a larger, multicentre sample will yield more consistent and informative results regarding the dysconnectivity in the brain of patients with schizophrenia. Moreover, we aim to explore the correlations between alterations in white matter tracts and clinical symptoms, as well as cognitive performance, to better understand the clinical relevance of our findings.

Furthermore, we applied the new method called harmonisation, which has recently been developed to integrate and utilise neuroimaging data from various sites (Mirzaalian *et al*., 2018; Karayumak *et al*., 2019), to merge many brain images from multiple locations by eliminating scanner-related differences. Although previous studies had calculated and combined *z*-scores of diffusion measures for joint analysis (Jahanshad *et al*., 2013; Kochunov *et al*., 2014; Kelly *et al*., 2018), they had a few limitations that *z*-scores might not capture the variance of the entire population and diffusion measures should have Gaussian distribution to use the *z*-score. By using training subsets from reference and target sites to determine inter-site differences, the harmonisation method can overcome the above mentioned limitations. It has been suggested that harmonisation has the advantage of allowing researchers to remove potential for scanner-related biases, while accounting for differences related to image acquisition parameters such as b value, spatial resolution, or gradient directions. We acknowledge that our sample size may not be as large as some other recent schizophrenia studies. However, we believe that the combination of probabilistic tractography, a larger sample size, and retrospective harmonisation will provide valuable insights into the white matter abnormalities in schizophrenia.

## Materials and methods

### Study participants

We combined brain images from four different studies: AMC, COBRE, NMorphCH, and UCLA. To achieve a homogeneous patient population, we only included subjects diagnosed with schizophrenia, excluding other schizophrenia spectrum disorders. Each study was approved by the applicable Institutional Review Board (IRB) and performed following the Declaration of Helsinki. The Asan Medical Center IRB approved the current study (IRB Number: 2021-0423).

#### Asan medical center (AMC)

Study participants were recruited from Asan Medical Center, Seoul, Korea. This dataset was previously approved by the Asan Medical Center Institutional Review Board (2012-0485). Patients were diagnosed with schizophrenia using the DSM-IV-TR, and healthy controls were those without any Axis I psychiatric diagnosis or any first-degree relative diagnosed with any Axis I psychiatric diagnosis. All subjects were between 20 and 40 years old, were right-handed, and did not have any organic disease affecting brain function. All brain images were acquired using a 3T scanner with an 8-channel SENSE head coil (Achieva; Philips Healthcare, Best, The Netherlands). Diffusion-weighted echo-planar imaging was conducted (TR[repetition time]/TE[echo time] = 5,422/70 ms; flip angle = 90°; field of view = 224 × 224 × 135 mm; and voxel size = 2 × 2 × 3 mm) for one baseline (*b* value = 0 s/mm ^ 2) and 32 gradient directions (*b* value = 1,000 s/mm ^ 2).

#### Center for biomedical research excellence (COBRE)

The Center for Biomedical Research Excellence (COBRE) project integrates multiple neuroimaging techniques with psychiatric, neuropsychological, and genetic testing. We obtained publicly available data from SchizConnect (http://schizconnect.org) (Wang *et al*., 2016). Imaging was conducted on a 3T scanner (Trio, Siemens Healthcare, Erlangen, Germany). Diffusion-weighted images were collected by a multiple-channel radiofrequency coil with GRAPPA(X2) (TE = 84 ms; TR = 9000 ms; number of excitations = 1; 72 slices with slice thickness = 2 mm; field of view = 256 × 256 mm; and matrix = 128 × 128) for 30 gradient directions (*b* = 800 s/mm ^ 2).

#### Neuromorphometry in schizophrenia by computer algorithm (NMorphCH)

The Neuromorphometry in Schizophrenia by Computer Algorithm (NMorphCH) project is a longitudinal study examining the clinical, cognitive, and neuroimaging data conducted at Northwestern University. We obtained publicly available data from SchizConnect (http://schizconnect.org). Imaging was conducted on a 3T scanner (Trio, Siemens Healthcare, Erlangen, Germany), and diffusion-weighted images were collected (TE = 86 ms; TR = 8000 ms; flip angle = 90°; matrix = 896 × 896; 35 slices with slice thickness = 2 mm) for 30 gradient directions (*b* = 0 and 800s/mm ^ 2).

#### UCLA consortium for neuropsychiatric phenomics LA5c study (UCLA)

The UCLA Consortium for Neuropsychiatric Phenomic LA5c study focused on understanding the dimensional structure of memory and cognitive control functions in healthy individuals and psychiatric patients(Gorgolewski *et al*., 2017). We obtained a publicly available dataset from OpenNeuro (https://openneuro.org). All participants were aged between 21 and 50 years old, without significant medical illness. Based on the Structured Clinical Interview for DSM-IV (SCID), patients were diagnosed with DSM-IV-TR. Imaging was conducted on a 3T scanner (Trio, Siemens Healthcare, Erlangen, Germany), and diffusion-weighted images were collected using an echo-planar sequence (TE = 93ms; TR = 9000ms; flip angle = 90°; matrix = 96 × 96; and slice thickness = 2 mm) for 64 directions (*b* = 1000s/mm ^ 2).

### Image processing and harmonisation

We used SliceDiffusionQC (https://github.com/pnlbwh/SlicerDiffusionQC) to eliminate bad gradient volumes for diffusion-weighted brain images. Specifically, each diffusion volume was categorised as good or bad based its distance to a median line, which was estimated from Kullback–Leibler divergence between consecutive diffusion volumes. Next, we conducted pre-processing, including axis alignment, centring, head motion, and eddy current correction through the Psychiatry Neuroimaging Laboratory (PNL) pipeline (https://github.com/pnlbwh/pnlutil).

We also implemented a retrospective harmonisation method to remove inter-site scanner-specific differences in diffusion-weighted brain images. We used the dMRIharmonisation (https://github.com/pnlbwh/dMRIharmonization) with default settings. As the UCLA dataset was designated as the reference dataset for harmonisation, age, sex, and handedness-matched subjects were selected from AMC, COBRE, and NMorphCH. We calculated the differences between the reference and target datasets by generating scale maps from rotation-invariant spherical harmonics feature templates. Then, harmonised data was obtained by applying scale maps to the target dataset. Overall image processing and harmonisation processes for diffusion-weighted brain images were described elsewhere in detail (Joo *et al*., 2021a).

### Probabilistic tractography

We applied the probabilistic tractography method to track white matter streamlines in the brain. Unlike the deterministic tractography, which assigns fixed direction at each voxel, the probabilistic tractography generates the distribution of the diffusion parameters at each voxel using Metropolis-Hastings Markov chain Monte Carlo sampling. We utilised the BEDPOSTX tool from the Diffusion Toolbox in the FMRIB software library (FSL) (Jenkinson *et al*., 2012). After that, we used the PROBTRACKX tool in FSL to generate a set of streamlines from the seed ROI to the target ROI. Five thousand samples were performed for each seed ROI. Then, we could estimate the degree of connectivity, or connection probability, as the number of streamline tracts from one seeding ROI that passed through a given target ROI divided by the total number of generated streamlines. The non-directional connection probability between two ROIs were defined by averaging the two probabilities obtained from tracking from each given ROI. The ROIs were determined according to the Desikan-Killiany atlas, and therefore there were 87 ROIs in each brain (Fischl *et al*., 2002).

### Symptoms and cognitive measures

Study participants from each dataset underwent standardised scales for measuring their psychiatric symptoms and cognitive functions. First, the symptom severity of patients was measured by the Positive and Negative Syndrome Scale (PANSS). Since the NMorphCH and UCLA datasets utilised the Scale for the Assessment of Positive Symptoms (SAPS) and Scale for the Assessment of Negative Symptoms (SANS) for assessing the symptom, instead of the PANSS, we converted these scores into PANSS scores using validated conversion equations for later analysis (Van Erp *et al*., 2014). Second, for cognitive measures, different cognitive measurement tools were used in each dataset. Thus, we only investigated commonly used few cognitive measures across different datasets: the vocabulary and block design subtests of the Wechsler Abbreviated Scale of Intelligence-II (WASI-II) or the Korean Wechsler Adult Intelligence Scale-IV (K-WAIS-IV), Word Fluency Test, and Trails Making Test A (TMT-A) or Color Trails Test part 1. These measures, respectively, represent visuospatial function, semantic language function, and executive function and have generally been suggested to be influenced by schizophrenia.

### Statistical analysis

After probabilistic tractography, we compared connection probabilities between patients with schizophrenia and healthy controls. We also compared demographic and clinical variables between patients and healthy controls. Independent *t* test, Mann–Whitney *U* test, and Chi-squared test were used to compare connection probabilities with demographic and clinical variables. We applied the Bonferroni correction method to correct multiple comparisons problems. Since it was argued that enhanced sensitivity of probabilistic tractography may counteract selectivity (Thomas *et al*., 2014), we tried to strictly control Type I error in testing the primary hypothesis that the connection probabilities estimated by probabilistic tractography were different between patients and healthy controls. In addition, we also evaluated the relationships between connection probabilities and symptoms or cognitive measures in the patient group using linear regression analysis and Bonferroni correction. Because cognitive measures could be affected by the age and sex of the study participants, we adjusted these variables during the regression analysis. All statistical analyses were performed using R (ver. 4.1.3) and Jamovi (ver. 2.3.21) (R Core Team, 2013, The Jamovi Project, 2021). Statistically significant *p* was set as < 0.05.

## Results

### Demographics and clinical characteristics

The final study sample consisted of 189 patients with schizophrenia and 213 healthy controls: 48 patients and 23 healthy controls for AMC; 57 patients and 73 healthy controls for COBRE; 40 patients and 32 healthy controls for NMorphCH; and 44 patients and 85 healthy controls for UCLA. The mean age of all patients with schizophrenia was 34.1 [9.93] years, which showed no statistically significant difference to 33.7 [10.1] years of controls (*U* = 19,660.0, *p* = 0.687). Of 189 patients and 213 healthy controls, 124 patients (65.6%) and 127 controls (59.6%) were male, and there was no significant difference in sex ratio (*χ* ^2^ = 1.530, *p* = 0.216). The mean total PANSS score was 63.9 [16.9] in the patients’ group. Specifically, the positive scale score was 17.1 [6.29], the negative scale score was 18.1 [6.19], and the general psychopathology scale score was 30.8 [8.05]. There was a significant difference in the age and sex of study participants between four different datasets (*χ* ^2^ = 30.7, *p* < 0.001; *χ* ^2^ = 29.1, *p* < 0.001). They also showed a significant difference in the mean PANSS score. Table 1 shows each dataset’s demographic and clinical characteristics and the final study sample in detail. Table 2 shows comparisons between four different datasets.

**Table 1.** Demographics and clinical characteristics of study participants

	Schizophrenia (*N* = 189)	Control (*N* = 213)	Statistics	*p*-value
***Demographics***				
Age (years)	34.1 [9.93]	33.7 [10.1]	19,660^†^	0.687
Sex (male, %)	124 (65.6)	127 (59.6)	1.53^‡^	0.216
***PANSS***				
Positive	17.1 [6.29]			
Negative	18.1 [6.19]			
General	30.8 [8.05] (*N* = 105)			
Total	63.9 [16.9] (*N* = 105)			
***Wechsler Intelligence Scale (score)***				
Vocabulary subtest	37.5 [15.8] (*N* = 125)	47.5 [10.6] (*N* = 127)	5.90^‡^	<0.001*
Block design subtest	36.1 [15.7] (*N* = 96)	50.3 [13.7] (*N* = 105)	6.80^‡^	<0.001*
*Word Fluency*	32.5 [12.4] (*N* = 135)	41.0 [10.1] (*N* = 112)	5.80^‡^	<0.001*
*Color Trails Test, part 1 (seconds)*	42.3 [20.7] (*N* = 173)	27.9 [10.9] (*N* = 195)	8033†	<0.001*

Mean [Standard deviation]; Number (Percentage).

† Mann–Whitney *U* test.

‡ Chi-squared test.

* Statistically significant *p*<0.05.

**Table 2.** Demographics and clinical characteristics of each dataset

	AMC		COBRE		NMorphCH		UCLA		*χ* ^2^	*p*-value
	SPR(*N* = 48)	CTR(*N* = 23)	SPR(*N* = 57)	CTR(*N* = 73)	SPR(*N* = 40)	CTR(*N* = 32)	SPR(*N* = 44)	CTR(*N* = 85)		
***Demographics***										
Age (years)	28.6 [6.22]	30.3 [5.15]	38.6 [12.5]	38.1 [12.0]	32.6 [6.74]	31.9 [8.54]	35.7 [8.87]	31.6 [8.53]	30.7	<0.001*
Sex (Male, %)	19 (39.6)	9 (39.1)	44 (77.2)	56 (76.7)	28 (70.0)	20 (62.5)	33 (75.0)	42 (49.4)	29.1	<0.001*
***PANSS***										
Positive	16.5 [7.46]	–	15.4 [5.19]	–	20.2 [6.80]	–	17.2 [4.69]	–		0.003*
Negative	16.6 [7.09]	–	17.8 [5.31]	–	21.8 [5.37]	–	16.6 [5.63]	–		<0.001*
General	28.9 [7.62]	–	32.3 [8.14]	–	–	–	–	–	4.38	0.036*
Total	62.0 [16.0]	–	65.5 [17.5]	–	–	–	–	–	1.05	0.305
***Wechsler Intelligence Scale (score)***										
Vocabulary subtest	32.0 [14.2]	46.4 [8.69]	–	–	54.7 [12.7]	63.8 [8.84]	31.5 [9.24]	44.1 [7.73]	74.5	<0.001*
Block design subtest	32.9 [11.3]	40.8 [9.99]	38.8 [18.3]	53.2 [13.3]	–	–	–	–	26.1	<0.001*
*Word Fluency*	29.2 [12.6]	38.7 [7.26]	33.7 [12.1]	41.8 [10.7]	35.1 [12.2]	40.5 [10.3]	–	–	10.8	0.005*
*CTT-1 (seconds)*	46.2 [24.5]	27.9 [8.56]	37.3 [18.7]	23.2 [7.16]	40.8 [15.7]	28.3 [11.8]	43.0 [17.5]	31.8 [12.3]	28.5	<0.001*

SPR, Schizophrenia; CTR, Control; CTT-1, Color Trails Test, part 1; AMC, Asan Medical Center; COBRE, Center for Biomedical Research Excellence; NMorphCH, Neuromorphometry in Schizophrenia by Computer Algorithm; UCLA, UCLA Consortium for Neuropsychiatric Phenomic LA5c study; Mean [Standard deviation]; Number (Percentage); Kruskal–Wallis H test was done between four different datasets.

* Statistically significant *p*<0.05.

### White matter tracts with probabilistic tractography

Since there were 87 ROIs, 3741 streamlines were generated during the tractography methods. We compared the probabilities of these streamlines between patients with schizophrenia and healthy controls. After Bonferroni correction for multiple comparisons, 11 different tracts showed statistically significant differences between patients with schizophrenia and healthy controls. First, tracts between the anterior corpus callosum and left caudate show significant differences (*U* = 25,995, Adjusted *p* = 0.002). The tracts between the anterior corpus callosum and both pallidum also showed significant differences (left: *U* = 25,704, Adjusted *p* = 0.006; right: *U* = 25,606, Adjusted *p* = 0.009). Moreover, the tract between left and right caudate also showed a significant difference (U = 25,861, Adjusted *p* = 0.003). In addition, the tract from left caudate to right pallidum and the tract from right caudate to left pallidum showed significant differences (*U* = 25,978, Adjusted *p* = 0.002; *U* = 25,734, Adjusted *p* = 0.005). The tract from right pallidum to both right caudal anterior cingulate and right pars opercularis also showed significant differences (*U* = 25,329, Adjusted *p* = 0.029; *U* = 25,565, Adjusted *p* = 0.011). Furthermore, the tract between right hippocampus and right superior temporal showed a significant difference (*U* = 25,252, Adjusted *p* = 0.039). Lastly, the tract from right paracentral to right postcentral and the tract from right ventral diencephalon to right parahippocampal showed significant differences (*U* = 25,412, Adjusted *p* = 0.021; *U* = 25,697, Adjusted *p* = 0.006). In all of these tracts, healthy controls exhibited stronger connection probabilities compared to patients with schizophrenia. Table 3 shows the comparisons of white matter tracts between patients with schizophrenia and healthy controls in detail.

**Table 3.** Comparison of connection probabilities between patients and controls

Tract	SPR (*N* = 189)	CTR (*N* = 213)	U	Adjusted *p*-value
Anterior corpus callosum-Left caudate	0.236 [0.163]	0.322 [0.171]	25,995	0.002*
Anterior corpus callosum-Left pallidum	0.069 [0.063]	0.103 [0.081]	25,704	0.006*
Anterior corpus callosum-Right pallidum	0.099 [0.082]	0.139 [0.092]	25,606	0.009*
Left caudate-Right pallidum	0.030 [0.028]	0.048 [0.041]	25,978	0.002*
Left caudate-Right caudate	0.110 [0.102]	0.167 [0.124]	25,861	0.003*
Left pallidum-Right caudate	0.025 [0.026]	0.039 [0.033]	25,734	0.005*
Right pallidum-Right anterior caudal cingulate	0.055 [0.040]	0.073 [0.045]	25,329	0.029*
Right pallidum-Right pars opercularis	0.075 [0.038]	0.090 [0.037]	25,565	0.011*
Right hippocampus-Right superior temporal	0.062 [0.045]	0.081 [0.049]	25,252	0.039*
Right parahippocampal-Right ventral diencephalon	0.051 [0.030]	0.069 [0.044]	25,697	0.006*
Right paracentral-Right postcentral	0.179 [0.058]	0.207 [0.056]	25,412	0.021*

SPR, Schizophrenia; CTR, Control; Mean [Standard deviation]; Mann–Whitney *U* test.

* Statistically significant Bonferroni-adjusted *p*<0.05.

### Relationship with symptoms and cognitive measures

We evaluated the relationships between connection probabilities of the 11 white matter tracts and symptoms or cognitive measures in patients with schizophrenia. As a result of linear regression analysis, we found that there was no significant relationship with the PANSS score after Bonferroni correction. Meanwhile, many tracts showed significant correlations for cognitive measures, even after adjusting for age and sex in regression models. For the vocabulary subtests, the tracts from the anterior corpus callosum to left caudate and right pallidum both showed significant correlations with the subtest scores (*β* = 16.8, Adjusted *p* = 0.003; *β* = 24.6, Adjusted *p* = 0.036). Moreover, the tract from left to right caudate and the tract from right paracentral to right postcentral also showed significant correlations (*β* = 25.2, Adjusted *p* = 0.004; *β* = 41.7, Adjusted *p* = 0.009).

In addition, nine out of 11 tracts showed significant relationships between the block design subtest scores. Three tracts, the tract between left pallidum and right caudate, the tract between left caudate and right pallidum, and the tract between right pallidum and right pars opercularis, showed much stronger correlations (|*β*| > 100.0) than others (*β* = 135.7, *p* = 0.012; *β* = 101.5, *p* = 0.017; *β* = 116.9, *p* < 0.001). Furthermore, the tract from left caudate to right caudate and the tract from right pallidum to right pars opercularis also showed significant correlations with the word fluency test score (*β* = 17.2, Adjusted *p* = 0.044; *β* = 66.9, Adjusted *p* = 0.006). The tract between right paracentral and right postcentral also showed significant correlation (*β* = 39.6, Adjusted *p* = 0.020).

Lastly, the Color Trails Test (CTT-1) scores showed a significant relationship with four tracts. The tracts from the anterior corpus callosum to left caudate and right pallidum both showed significant correlations (*β* = −15.4, Adjusted *p* = 0.013; *β* = −25.1, Adjusted *p* = 0.049). Moreover, the tract from left to right caudate and the tract from right paracentral to right postcentral also showed significant correlations with the CTT-1 scores (*β* = −20.6, Adjusted *p* = 0.032; *β* = −41.8, Adjusted *p* = 0.024). Table 4 shows the correlations between brain tract connectivity and symptoms or cognitive measures in detail.

**Table 4.** Correlations of connection probabilities to symptoms and cognitive measures in patients

Symptoms and cognitive measures/White matter tract	*β*	Adjusted *p*-value
*Vocabulary subtest (score)*		
Anterior corpus callosum-Left caudate	16.8	0.003*
Anterior corpus callosum-Right pallidum	24.6	0.036*
Left caudate-Right caudate	25.2	0.004*
Right paracentral-Right postcentral	41.7	0.009*
*Block design subtest (score)*		
Anterior corpus callosum-Left pallidum	65.5	<0.001*
Anterior corpus callosum-Right pallidum	41.5	0.015*
Anterior corpus callosum-Left caudate	34.6	<0.001*
Left caudate-Right caudate	44.5	<0.001*
Left caudate-Right pallidum	101.5	0.017*
Left pallidum-Right caudate	135.7	0.012*
Right pallidum-Right pars opercularis	116.9	<0.001*
Right paracentral-Right postcentral	78.6	<0.001*
Right parahippocampal-Right ventral diencephalon	84.5	0.048*
*Word Fluency*		
Left caudate-Right caudate	17.2	0.044*
Right pallidum-Right pars opercularis	66.9	0.006*
Right paracentral-Right postcentral	39.6	0.020*
*Color Trails Test, part 1 (seconds)*		
Anterior corpus callosum-Left caudate	−15.4	0.013*
Anterior corpus callosum-Right pallidum	−25.1	0.049*
Left caudate-Right caudate	−20.6	0.032*
Right paracentral-Right postcentral	−41.8	0.024*

PANSS, Positive and Negative Syndrome Scale; *β*, beta coefficient in a regression model controlled for age and sex.

* Statistically significant Bonferroni-adjusted *p*<0.05.

## Discussion

In this study, we performed probabilistic tractography using diffusion MRI data to investigate the alterations of brain white matter tracts in patients with schizophrenia, overcoming the limitations of deterministic tractography methods. Furthermore, we significantly enhanced the statistical power of our findings by merging datasets from multiple study sites to create a large, combined dataset. By utilising the harmonisation method, we were able to adjust for inter-site image variabilities stemming from scanner-related differences, rather than simply merge disparate datasets.

Our results revealed significant differences in multiple brain white matter tracts between patients with schizophrenia and healthy controls. First of all, we observed significant differences in several tracts related to anterior corpus callosum. The corpus callosum, a major commissural pathway between the cerebral hemispheres, has been a highly interesting region in schizophrenia research (Arnone *et al*., 2008; Patel *et al*., 2011) due to its relationship with the severity of psychotic symptoms. Patients with schizophrenia have previously shown structural (Keshavan *et al*., 2002; Rao *et al*., 2011) and diffusional abnormalities (Whitford *et al*., 2010) in the corpus callosum, indicative of impaired inter-hemispheric connectivity. Moreover, tracts associated with the pallidum and caudate displayed significant differences between patients and controls. While findings about anatomical alterations have sometimes been inconsistent, cortico-striatal streamlines have often been reported as altered in patients with schizophrenia. These alterations are associated with hypokinesia (Bracht *et al*., 2013), cognitive impairment mediated by an impaired default mode network (Huang *et al*., 2018), and both positive and negative symptoms of schizophrenia (McCutcheon *et al*., 2019). In addition, we identified significant differences in tracts related to other brain regions, such as pars opercularis, hippocampus, and superior temporal areas. Despite occasional inconsistencies in research findings, some studies suggest that these regions exhibit decreased volume or reduced cortical thickness in schizophrenia (Kasai *et al*., 2003; Harrison, 2004, Knøchel *et al*., 2016). In particular, structural alterations in the superior temporal lobe or gyrus, especially progressive volume reduction, are emphasised in the pathophysiology of schizophrenia (Sun *et al*., 2009; Takahashi *et al*., 2009).

Among these white matter tracts, we identified significant relationships between various white matter tracts and cognitive measures. First, the language function, as measured by the vocabulary subtest, was associated with the caudate and corpus callosum. Furthermore, the pallidum and pars opercularis showed relationships with word fluency test scores. Previous studies have suggested that these regions play crucial roles in language control and are altered in patients with schizophrenia (Friederici, 2006; Li *et al*., 2015, Trëhout *et al*., 2017). Notably, the pars opercularis is part of the well-known language-related brain region, Broca’s area. The block design subtest score, which measures visuospatial and executive functions, also showed significant relationships with numerous white matter tracts. It is widely recognised that executive function is impaired in schizophrenia (Orellana and Slachevsky, 2013). Lastly, the CTT-1 scores showed significant relationships with certain regions. Although the corpus callosum has been implicated in attention and processing speed (Hinkley *et al*., 2012), the relationships between other regions and these cognitive processes remain insufficiently explored. Further research on these relationships are recommended.

On the other hand, we did not identify a significant relationship between psychiatric symptoms, as measured by the PANSS score, and white matter tracts. Given that prior research has reported associations between alterations in white matter tracts and symptoms of schizophrenia, this finding was somewhat unexpected. This discrepancy might be due to the conservative nature of our multiple comparisons correction or to the heterogeneity of our study participants. Furthermore, we did not account for whether participants were in acute or remission phases, potentially leading to a wide variation in symptoms. Another potential confounding factor we did not consider is the use of antipsychotic medications in the correlation analysis between the PANSS score and white matter tracts.

Our study has several limitations. As mentioned above, we did not account for certain demographic and clinical variables, including ethnicity, current clinical status, and illness duration, while conducting statistical analysis. For instance, the open dataset participants were primarily Caucasian, while all participants in the AMC dataset were Asian. Thus, there were ethnic differences within the merged dataset. Nevertheless, we performed retrospective harmonisation to mitigate inter-site differences in brain images, which could also likely attenuate this difference. Moreover, we were unable to examine the potential influence of antipsychotics due to data unavailability. While some studies have recommended considering medication when investigating DTI (Karlsgodt, 2016), several other studies have found no significant relationship between diffusion measures and medication (Kanaan *et al*., 2009; Koshiyama *et al*., 2020). Lastly, we did not compare the relationships between connection probabilities and other clinical features such as intellectual ability, education attainment, or socioeconomic status. As some previous studies have suggested associations between these factors and white matter integrity (Penke *et al*., 2012; Noble *et al*., 2013; Shaked *et al*., 2019), further research incorporating more detailed demographic information is required.

In conclusion, our study observed significant alterations in white matter tracts within the brains of patients with schizophrenia. Even after applying a conservative multiple comparisons correction method, numerous brain tracts demonstrated significant differences between patients with schizophrenia and healthy controls. Importantly, several of these tracts exhibited significant correlations with cognitive measures, suggesting that these tracts might play a role in the pathophysiology of schizophrenia. Future research is encouraged to examine these brain regions more specifically and to investigate their involvement in the progression of schizophrenia. Longitudinal studies could also provide valuable insights into the dynamic changes in white matter tracts over time and their potential impact on symptom severity and cognitive functioning. Building on these findings, future research can contribute to a deeper understanding of the underlying neural mechanisms of schizophrenia.
